# ENCD: a manually curated database of experimentally supported endocrine system disease and lncRNA associations

**DOI:** 10.1093/database/baac113

**Published:** 2023-01-19

**Authors:** Ming Hao, Yue Qi, Rongji Xu, Kangqi Zhao, Mingqing Li, Yongyan Shan, Tian Xia, Kun Yang, Wuyang Hasi, Cong Zhang, Daowei Li, Yi Wang, Peng Wang, Hongyu Kuang

**Affiliations:** College of Bioinformatics Science and Technology, Harbin Medical University, 194 Xuefu Road, Harbin 150081, China; College of Bioinformatics Science and Technology, Harbin Medical University, 194 Xuefu Road, Harbin 150081, China; Department of Endocrinology, The First Affiliated Hospital of Harbin Medical University, 23 Youzheng Road, Harbin 150081, China; Department of Endocrinology, The First Affiliated Hospital of Harbin Medical University, 23 Youzheng Road, Harbin 150081, China; Department of Endocrinology, The First Affiliated Hospital of Harbin Medical University, 23 Youzheng Road, Harbin 150081, China; Department of Endocrinology, The First Affiliated Hospital of Harbin Medical University, 23 Youzheng Road, Harbin 150081, China; Department of Endocrinology, The First Affiliated Hospital of Harbin Medical University, 23 Youzheng Road, Harbin 150081, China; Department of Endocrinology, The First Affiliated Hospital of Harbin Medical University, 23 Youzheng Road, Harbin 150081, China; Department of Endocrinology, The First Affiliated Hospital of Harbin Medical University, 23 Youzheng Road, Harbin 150081, China; Department of Endocrinology, The First Affiliated Hospital of Harbin Medical University, 23 Youzheng Road, Harbin 150081, China; Department of Endocrinology, The First Affiliated Hospital of Harbin Medical University, 23 Youzheng Road, Harbin 150081, China; College of Bioinformatics Science and Technology, Harbin Medical University, 194 Xuefu Road, Harbin 150081, China; Department of Endocrinology, The First Affiliated Hospital of Harbin Medical University, 23 Youzheng Road, Harbin 150081, China; Department of Endocrinology, The First Affiliated Hospital of Harbin Medical University, 23 Youzheng Road, Harbin 150081, China

## Abstract

ENCD (http://www.bio-server.cn/ENCD/) is a manually curated database that provides comprehensive experimentally supported associations among endocrine system diseases (ESDs) and long non-coding ribonucleic acid (lncRNAs). The incidence of ESDs has increased in recent years, often accompanying other chronic diseases, and can lead to disability. A growing body of research suggests that lncRNA plays an important role in the progression and metastasis of ESDs. However, there are no resources focused on collecting and integrating the latest and experimentally supported lncRNA–ESD associations. Hence, we developed an ENCD database that consists of 1379 associations between 35 ESDs and 501 lncRNAs in 12 human tissues curated from literature. By using ENCD, users can explore the genetic data for diseases corresponding to the body parts of interest as well as study the lncRNA regulating mechanism for ESDs. ENCD also provides a flexible tool to visualize a disease- or gene-centric regulatory network. In addition, ENCD offers a submission page for researchers to submit their newly discovered endocrine disorders-genetic data entries online. Collectively, ENCD will provide comprehensive insights for investigating the ESDs associated with lncRNAs.

**Database URL** http://www.bio-server.cn/ENCD

## Introduction

The endocrine system is an important system that secretes various hormones and is involved with the nervous system in regulating the body’s metabolism and ensuring proper communication between organs, which is essential for maintaining a constant internal environment (1). Normally, the hormone levels in the human body are balanced. However, various multidimensional factors can disturb the physiological balance of our hormones, resulting in high or low levels of hormones in the body and a range of endocrine system diseases (ESDs) (2). For example, diabetes as the most common disease of the endocrine system is now the fifth leading cause of death in the world and will increase to 643 million by 2030 and to 783 million by 2045 (3). Autoimmune thyroid disease not only carries a risk of cancer but also has a high incidence, affecting 5% of the population (4). The underlying causes of ESDs are complex and have multiple levels, including hereditary factors (genome, transcriptome and epigenetic mechanisms) and environmental factors (5). Therefore, one of the biggest challenges in ESD research is to clarify molecular mechanisms of the disease.

Long non-coding RNAs (lncRNAs) are a family of non-coding RNA molecules longer than 200 nucleotides and have key roles in gene regulation (6, 7). Emerging genomic technologies have shown that lncRNAs can regulate cell fate and modulate endocrine processes (8, 9). A number of experimentally supported lncRNA–disease association databases, such as LncRNADisease (10), NONCODE (11), LncACTdb (12), LnCeCell (13) and LnCeVar (14), predicting the relationship between human lncRNAs and diseases have emerged. However, there are no resources with experimental support and recent data to specifically investigate the relationship between ESDs and lncRNAs. Therefore, to fill this gap and provide higher-quality and more comprehensive resources to ESD researchers, we developed ENCD, a manually curated database of experimentally supported ESD–lncRNA associations.

## Materials and methods

### Data acquisition and curation

In order to ensure the high quality of the database, we referred to the data collation steps described in lnc2cancer (15), NSDNA (16) and GABC (17). ENCD aims to collect and integrate information on all lncRNAs associated with endocrine diseases, and its data sources are broadly divided into two categories. One category is derived from other databases, such as NONCODE (11), Gencards (18), Ensembl (19), HGNC (20) and Genbank (21). The other category was derived from already published studies and all literature related to endocrine disorders in recent years. To collect lncRNA–disease associations, a list of keywords, such as ‘long non-coding RNA’, ‘diabetes’, ‘pituitary dwarf’ and ‘multiple endocrine neoplasia type 1’, was queried based on the PubMed database (22). We collected detailed information of lncRNA–disease associations, including official names, synonyms, gene IDs, sequence and positional information of the lncRNAs, experimental techniques (e.g. microarray and quantitative-Real-Time-PCR (qRT-PCR)), regulating directions (up- or down-expression of lncRNAs), literature information (PubMed ID, year of publication and title of paper) and a brief functional description of lncRNA–disease associations from the original studies. According to relationships between experimental types and data, the types of disease–lncRNA associations were grouped into four categories, including strong evidence, weak evidence, direct evidence and high-throughput. This strategy has been used to mine miRNA–target (23) and cancer–lncRNA (15) associations. Strong evidence refers to the evidence of the correlation between lncRNAs and diseases obtained through qRT-PCR, RNA interference, *in vitro* knockdown and other laboratory technologies. Weak evidence refers to the prediction of disease candidate lncRNAs through microarray and machine-learning methods. Direct evidence refers to a direct interaction between lncRNA and disease phenotypes through experiments. High-throughput refers to the usage of high-throughput sequencing technology and bioinformatics prediction tools to analyse lncRNA functions.

**Figure 1.**
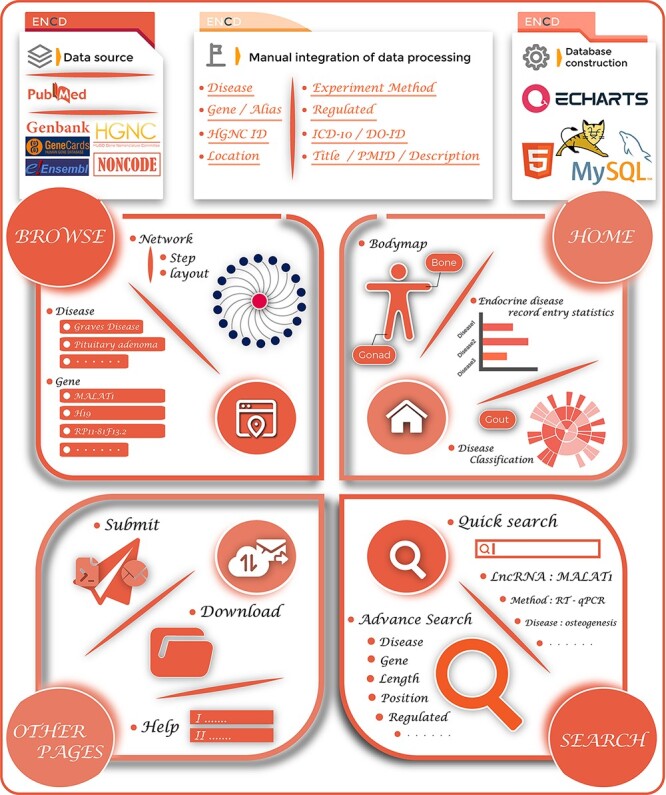
The overview of the ENCD database. The top panel illustrates the building process of ENCD, including the data source, data mining pipeline and database construction. The bottom panel illustrates the functions and interfaces of ENCD. The HOME interface provides a bodymap and statistics of data. The BROWSE and SEARCH interfaces provide different functions to query data from ENCD. The Submit, Download and Help interfaces are shown as OTHER PAGES.

During data integration, we retrieved the names of endocrine diseases as well as their ICD-10/DO-ID numbers. For lncRNAs, we collected the gene name, gene alias, ENSEMBLE ID, HGNC ID, gene location, gene length and direction of their disease regulation. For literature data entries from the PubMed database, we provided hyperlinks to original articles, PMID, year of publication, title and a brief functional description of the study. Furthermore, ENCD also classified 35 ESDs into 12 groups based on their location of human organs/tissues to further refine the categorization of different diseases. As a result of the manual screening and integration, 1379 data items were stored in the ENCD. The complete ENCD building process is shown in Figure 1.

### Database construction

ENCD is a web-based database, where data are stored and managed using MySQL, a freely accessible data management system (https://www.mysql.com/). The web interface was built using JAVA Server Pages (https://www.java.com/). The scripts for the data processing programs were written in JAVA. The web service is run on the Apache Tomcat web server. ENCD also supports the current major browsers (such as Microsoft Edge, Google Chrome, Firefox and Safari) and is available free of charge from http://www.bio-server.cn/ENCD/.

## Results

### ENCD database content

**Figure 2.**
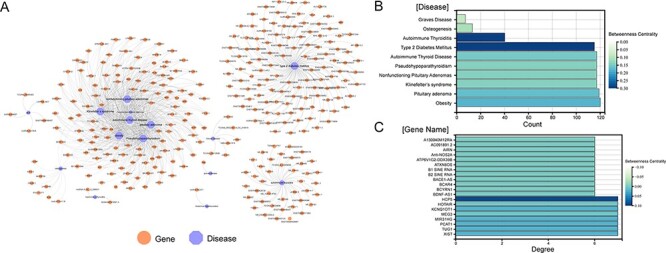
Visualization of all gene**-**disease associations in ENCD. (A) A network diagram constructed for all gene-diseases in ENCD, showing all disease and gene names in ENCD. (B) The betweenness centrality of endocrine diseases in the network. (C) The betweenness centrality of lncRNAs in the network.

A total of 1379 associations between 35 ESDs and 501 lncRNAs were manually curated after systematically reviewing hundreds of published articles. To facilitate the presentation of relationships between diseases and genes, network diagrams of all associations were constructed (Figure 2A). Diseases (autoimmune thyroiditis and Type 2 diabetes mellitus), as well as gene (*HCP5*), had a higher betweenness centrality, demonstrating that these nodes have more control over the ESD–lncRNA network through the statistics of the network diagram (Figure 2B and C). Previous research identified that *HCP5* provides a novel epigenetic mechanism for premature ovarian insufficiency pathogenesis by regulating MSH5 transcription and deoxyribonucleic acid (DNA) damage repair via the interaction with YB1, providing a novel epigenetic mechanism for premature ovarian insufficiency pathogenesis (24). We collected gene names, gene aliases, ENSEMBLE IDs, HGNC IDs, gene locations, gene lengths and associations with diseases for all lncRNAs in the network. We also collected the disease name, ICD-10/DO-ID number, corresponding experimental method of the article species, article title, PMID, year of publication of the article and a detailed functional description of the gene-disease relationship. By analysing the topological nature of the nodes in the network, it is possible to uncover the specificities and similarities in the way genes play a regulatory role and to draw analogies between the similarities of different diseases. In addition, in order to classify the accuracy of the data, we added the correlation between experiments and data, which can be classified into four categories overall: strong evidence, weak evidence, direct evidence and high-throughput (Materials and methods). Also, the lncRNA–disease associations were grouped based on their location of human organs/tissues. Users can get detailed classification information on these diseases on the homepage.

## User interface

### Homepage

To make it easier for researchers to navigate queries, ENCD provides a user-friendly web interface and multiple ways to access the data. To visit other pages of the ENCD database, users can click on the navigation bar above for quick access. We also added a Quick Search function in the upper navigation bar. By clicking the magnifying glass icon in the upper right corner to bring up the Quick Search interface, users can quickly search the database for all entries containing the keywords by clicking on the examples below the search bar or manually entering the keywords such as gene name, disease name or experimental method. On the homepage, ENCD provides users various charts, including (i) Bodymap: users can click on the corresponding location/position name of the bodymap to view the corresponding disease–gene data in that location, (ii) Disease Classification Sunburst Chart: this chart details the diseases in different locations. Users can click on the corresponding disease name in the outer circle to view all the corresponding disease data and (iii) Disease Number Statistical Chart: this chart records the number of entries containing the disease, and clicking on the bar chart can view detailed information.

### Browsing

ENCD provides a powerful browsing interface to make it more intuitive for users to navigate lncRNA–disease associations. In the Browse interface, users can choose to build a disease-centric or gene-centric regulatory network. On the left side of the browse, ENCD provides two lists of diseases/genes and users can search for relevant disease–gene pairs by clicking on the corresponding entries. The results of this query are visualized on the right side of the browse page using a network diagram developed using the Charts plug-in software, which allows users to click on different entries in the list on the right side to build different networks. By clicking on the corresponding disease/gene node in the network diagram, users can query all data entries that contain the name of that node. In the middle of ‘Network Tools’, the Keywords row shows which node the network diagram is built from. Also, for this network diagram, ENCD provides a custom reorganization of the network, where users can set the network step and network layout and click the Submit button to rebuild the network. The constructed network diagram can be saved locally by clicking on the Download button above. The construction of gene-disease regulatory networks can better reveal the regulatory relationships between genes and diseases. Studying the regulatory networks can identify the co-regulatory relationships of multiple genes. Increasing the network step can uncover the central genes that play a regulatory role in multiple diseases, and studying such genes can lead to a better understanding of the pathogenesis of endocrine diseases.

### Search function

In the Search interface, we provide two types of queries: (i) Quick Search: same as the Quick Search interface on the homepage, users can use the Quick Search tool to query all entries in the database containing a certain keyword by entering it; (ii) Advanced Search: in the Advanced Search, users can set more stringent conditions to filter the query results. All input fields are optional, and we provide a variety of filtering criteria in this function. In addition to the usual gene/disease keyword query, ENCD provides chromosome location, the direction of gene regulation for the disease, gene length and experimental type. By providing a combination of search operators, users can precisely select the data they are interested in. In addition, all query results can be downloaded in different formats by clicking on the Save button above the results table.

### Submit, Download and Help

The ENCD database welcomes users to submit their newly discovered endocrine disorders-genetic data entries online. Users can save their submissions as doc/txt/xlsx data along with the user’s contact details and submit them to ENCD. Upon receipt of a new submission, we will carry out manual verification and check the paper’s content. Once we have confirmed the validity of the data, we will update our database and inform the uploader as soon as possible. In addition, a download interface is available for users to access all manually collected endocrine disease-related data in the ENCD by clicking on the ‘Download’ folder. If users have any questions about ENCD, they can click on the Help button in the navigation bar to access the Help screen, which provides explanations and tutorials for some common problems. Further, users can submit questions via the ‘Other Questions’ function on the ‘Submit’ screen or directly contact us using the contact details provided on the ‘Contact us’ page.

### User utility

**Figure 3.**
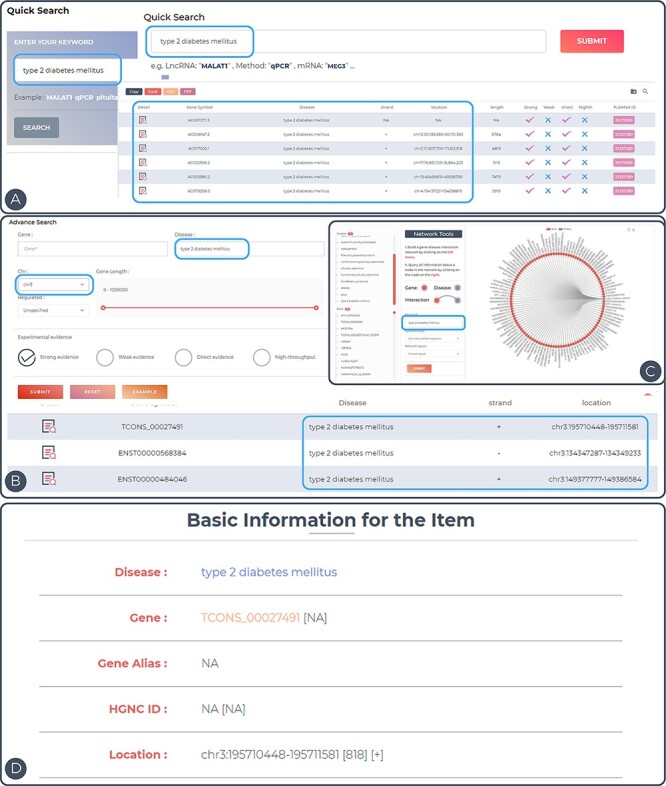
Features and utilities of ENCD. (A) A single keyword search and the corresponding results presentation using a quick query. (B) A multi-criteria search and display of results using an advanced query. (C) Browsing the web based on keywords in the interface. (D) Detailed display of query entries.

To demonstrate the utility of ENCD, the disease ‘type 2 diabetes mellitus’ was used as an example (Figure 3A–D). Type 2 diabetes mellitus (T2DM) is a common clinical chronic disease, with the global prevalence of diabetes in adults increasing from 4.7% in 1980 to 8.5% in 2014. T2DM affects 90–95% of adults with the disease, and the pathogenesis of this disease has not yet been established. Upstream and downstream analyses of the genes involved in T2DM may provide new insights into the pathogenesis in the future (25). All entries in the database can be accessed directly by entering the keyword ‘type 2 diabetes mellitus’ in the Quick Search page (Figure 3A). In the Search interface, ‘type 2 diabetes mellitus’ was inputted in the Disease search field and chromosome 3 was selected in the chromosome position (Figure 3B). Only the entries related to this keyword on chromosome 3 will be displayed in the results. In the Browse interface, users can click on the entry for ‘type 2 diabetes mellitus’ in the disease column on the left side and a network diagram of ‘type 2 diabetes mellitus’ and all related genes will be displayed on the right side (Figure 3C). The details of the entry can be viewed by clicking on the Detail icon to the right of the query result (Figure 3D). In the detail page, the disease–gene network function was performed by generating an lncRNA-centric network and a disease-centric network. Based on this construction, users can start a new search for related lncRNAs or diseases.

## Discussion and conclusion

The endocrine system regulates metabolic processes in the body. Alterations in the hormone-secreting endocrine system associated with various factors such as behavioural problems, metabolic syndrome and bone and immune disorders can cause organ dysfunction and disease states (26). LncRNAs are crucial to the homeostasis of the endocrine system as they affect endocrine gland functions (27) and participate in the formation of endocrine gland–associated tumours (28).

To the best of our knowledge, the high quality of ESD–lncRNA relationships is not well documented in any databases. With the rapid development of high-throughput sequencing, there has been increasingly more attention on lncRNA–disease associations with multiple databases developed collecting relevant lncRNAs as previously described. Currently, there are a few databases focusing on exploring lncRNA functions based on different analysis strategies (29–32). To provide more comprehensive information of lncRNAs, we have added more links to other lncRNA databases, including LncBook 2.0 (33), LncRNAwiki 2.0 (34) and LnciPedia (35). However, our current study is limited by the scope of the datasets. For example, the significant growth in single-cell omics data makes it particularly important to identify disease markers at the single-cell level (36). In addition, the epigenetic aspect of lncRNAs is also widely used to identify molecular markers of the disease (37). These aspects of the datasets are not included in our database. In the future, we will continuously update ENCD by integrating more datasets and functional tools. In conclusion, ENCD can be considered as a promising tool for the study of ESDs.

## Data Availability

All data used in the analysis can be obtained at http://www.bio-server.cn/ENCD/.
